# The use of intraoperative CT-neuronavigation in Wiltse approach. A technical note

**DOI:** 10.3389/fsurg.2024.1433273

**Published:** 2024-09-02

**Authors:** Marco Battistelli, Federico Valeri, Manuela D’Ercole, Alessandro Izzo, Alessandro Rapisarda, Filippo Maria Polli, Nicola Montano

**Affiliations:** ^1^ Department of Neuroscience, Neurosurgery Section, Università Cattolica del Sacro Cuore, Rome, Italy; ^2^ Department of Neurosurgery, Fondazione Policlinico Universitario Agostino Gemelli IRCCS, Rome, Italy

**Keywords:** Wiltse approach, CT-neuronavigation, navigation, spine surgery, disc herniation, spine schwannoma

## Abstract

**Introduction:**

The paraspinal approach was first introduced in 1968 and later refined by Leon Wiltse to gain access to the lateral interevertebral foraminal region. However, challenges can arise due to unfamiliarity with this approach, unique patient anatomy, or in case of revision surgery, potentially elevating the risk of complications and/or poor outcome.

**Methods:**

Here we report on two cases in which the intraoperative Oarm CT neuronavigation was used during a Wiltse approach. Under general anesthesia, the spinous process near the surgical level is exposed through a midline incision. The patient's reference anchor is then attached to the exposed spinous process. Intraoperative CT is acquired and transferred to the Stealth Station S8 Surgical Navigation System (Medtronic). The Wiltse approach is now performed through a paramedian incision under neuronavigation guidance and perfectly tailored to the patient's unique anatomy.

**Results:**

The first case was a patient harboring a left lumbar intraextraforaminal schwannoma and the second one was a patient with an extraforaminal lumbar disc herniation at the adjacent level of a previous lumbar instrumentation. We were able to easily identify and remove both the lesions minimizing the surgical approach with no complication and optimal clinical outcome.

**Discussion and Conclusion:**

Our cases demonstrate the feasibility of application of intraoperative O-arm CT-neuronavigation to the Wiltse approach. In our opinion, this technique helps in minimizing the surgical approach and rapidly identifying the lesion of interest. Further studies are needed to address the effective utility and advantages of intraoperative CT-neuronavigation in this specific surgical scenario.

## Introduction

The paraspinal posterolateral approach, also known as the Wiltse approach, consists of the exposure of the facet joint-transverse process junction. This is obtained through a blunt and no traumatic separation of medial multifidus and lateral longissimus muscles. This approach is used for the treatment of various pathologies located in the foraminal and/or extraforaminal area, both degenerative and oncological ones (1). The paraspinal approach presents several limitations such as the need for familiarity with anatomy, poor visibility, tendency to lateralization from the entry point with consequent risk of root injury (2). In this perspective, CT-guided neuronavigation might be useful in determining real-time precise regional anatomy thus allowing a tailored paraspinal approach based on patients’ specific anatomy with respect to distortions due to pathology. The present technical note report on two cases in which CT-guided neuronavigation was applied to paraspinal posterolateral Wiltse approach. We describe the surgical technique, the outcome of patients and discuss the pertinent literature.

## Methods

### Patients

Two patients underwent the intraoperative CT-neuronavigated Wiltse approach. The first one describes its application in the removal of an intra-extraforaminal lumbar spine tumor, while the second one highlights its value in lumbar extraforaminal disc herniation in a level adjacent to a previous pedicle-screw stabilization. Approval from an ethical committee was not required as the procedure adhered to the principles of good clinical practice.

### Surgical technique

Following the administration of general anesthesia, the patient is placed prone on a radiolucent table on iliac crests and chest supports. A midline incision is made, followed by sub-periosteal dissection of the spinous process near the surgical level. The patient's reference anchor is attached to this spinous process. Intraoperative CT is then acquired and transferred to the Stealth Station S8 Surgical Navigation System (Medtronic). A paramedian skin incision, the opening of the fascia, and dissection of the multifidus-longissimus muscles areolar tissue are carefully tailored and executed with the neuronavigation guidance. Under microscope magnification, the lesion targeted for removal is approached with navigation probe guidance, ensuring proper exposure. The extent of this exposure is determined using the navigation system.

## Results

### Case presentation

#### Case 1

**Figure 1:**
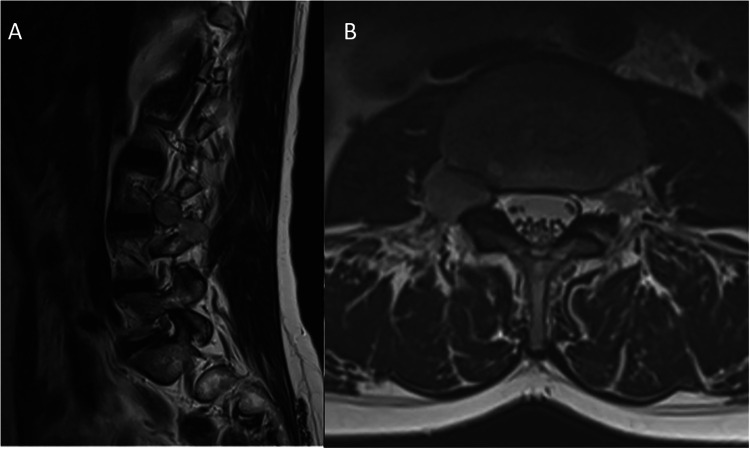
Pre-operative T2-WI sagittal **(A)** and axial **(B)** spine MRI. It can be noted the later extension toward the iliopsoas muscle (Asazuma type IIc) which appears as a hyperintense intraforaminal dumbbell shaped lesion.

**Figure 2:**
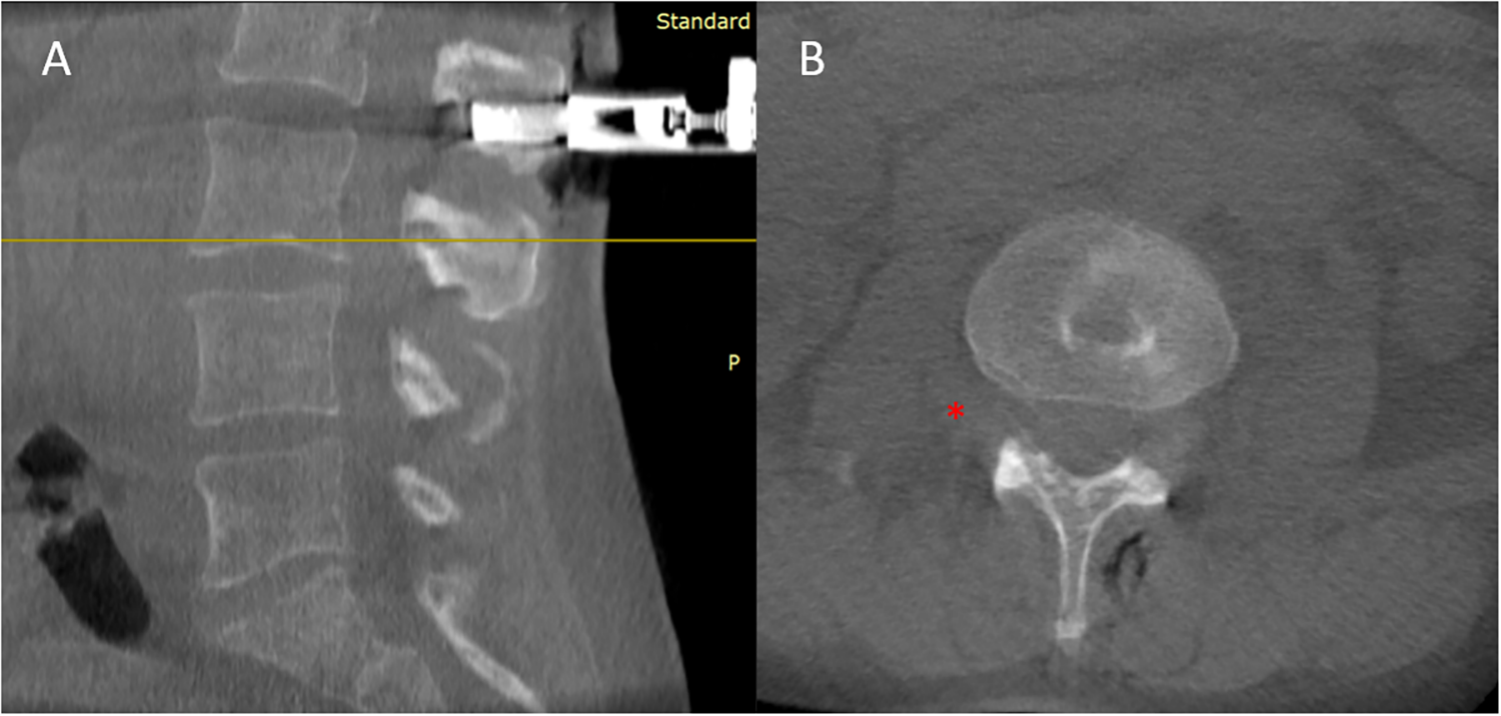
Intraoperative **(A)** sagittal and **(B)** axial CT-scan. The lesion (red asterisk) appears as a hypodensity lateral to the right L3–L4 foramen which extends beneath ipsilateral psoas muscle.

**Figure 3:**
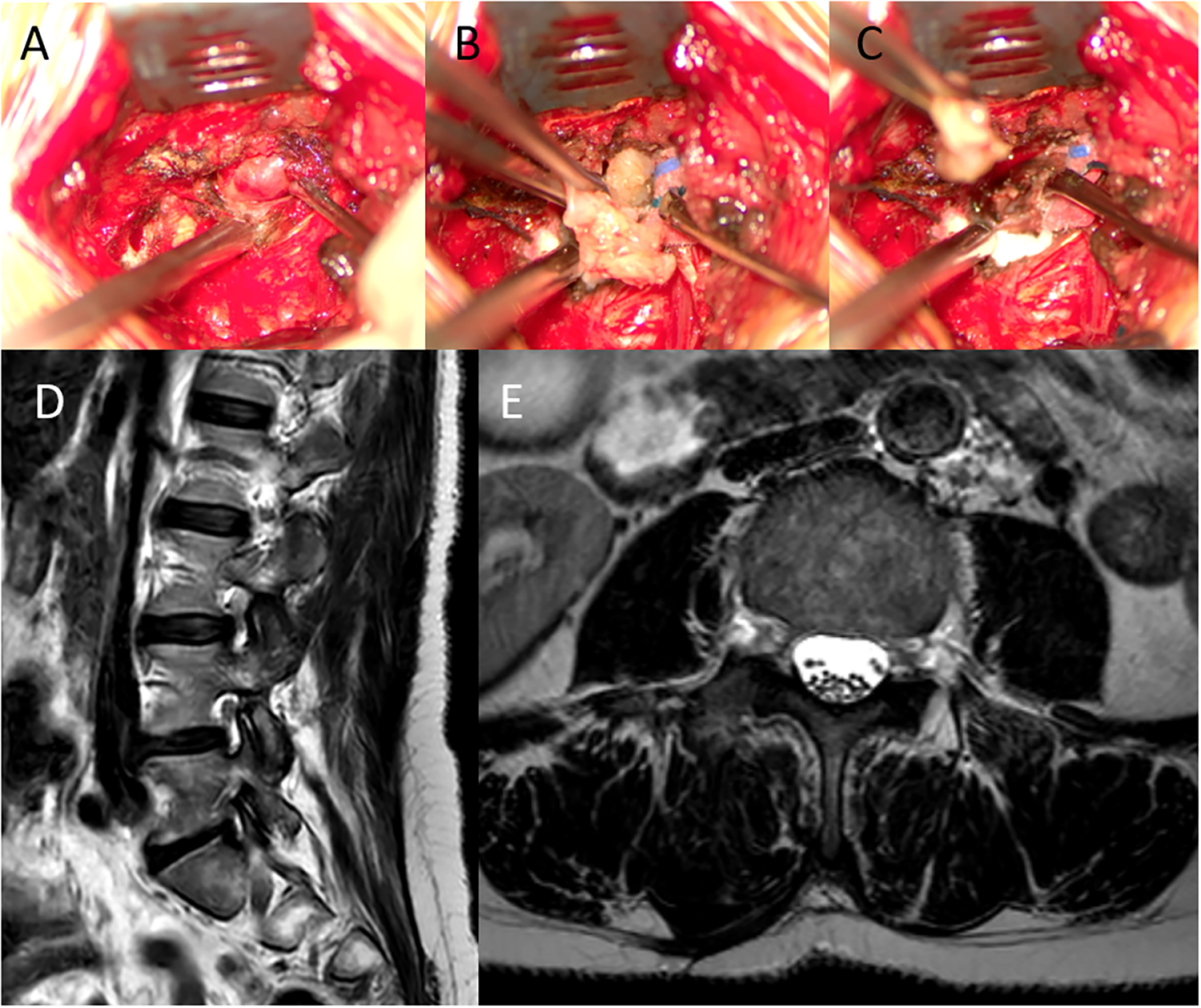
Intraoperative view **(A, B, C)** and follow-up MRI **(D, E)**. Tumor was approached via O-Arm neuronavigated Wiltse approach. **(A)** Exposure of the tumor. **(B)** Opening of the capsule. **(C)** Schwannoma removal. It can be appreciated how skin incision and soft tissue dissection in perfectly centered over the lesion. **(D)** Sagittal and **(E)** axial T2 WI 6-months follow-up spine MRI showing the gross total tumor resection.

A 54-year-old female patient was admitted with a 3-years history of right sciatic pain. At neurological examination, the patient exhibited thermic, tactile, and pain hypoesthesia in the right L3 innervation territory. Spine magnetic resonance imaging (MRI) showed a welldefined lesion, Asazuma grade IIc (3), extending from the right L3–L4 foramen towards the paraspinal muscles highly suggestive for schwannoma (Figure 1). Surgery was performed under general anesthesia and intraoperative neurophysiological neuromonitoring. After placing a patient-anchored reference on the L2 spinous process, an intraoperative CT scan was obtained for navigation (Figure 2). The lesion was identified laterally to the right L3–L4 apophyseal joint, just between the transverse processes of the two vertebrae (Figure 2) and the skin incision was placed accordingly. Under neuronavigation control a Wiltse approach was made and the tumor was reached with a blunt dissection and removed “en bloc” (Figures 3A–C). The operative time was 87 min and blood loss 100 ml, respectively. Histological examination confirmed the diagnosis of schwannoma. Postoperative course was uneventful, with the disappearance of radicular pain. The patient was discharged 2 days after surgery. At 6-months follow-up, she was asymptomatic with no sign of recurrence (Figures 3D,E).

#### Case 2

**Figure 4:**
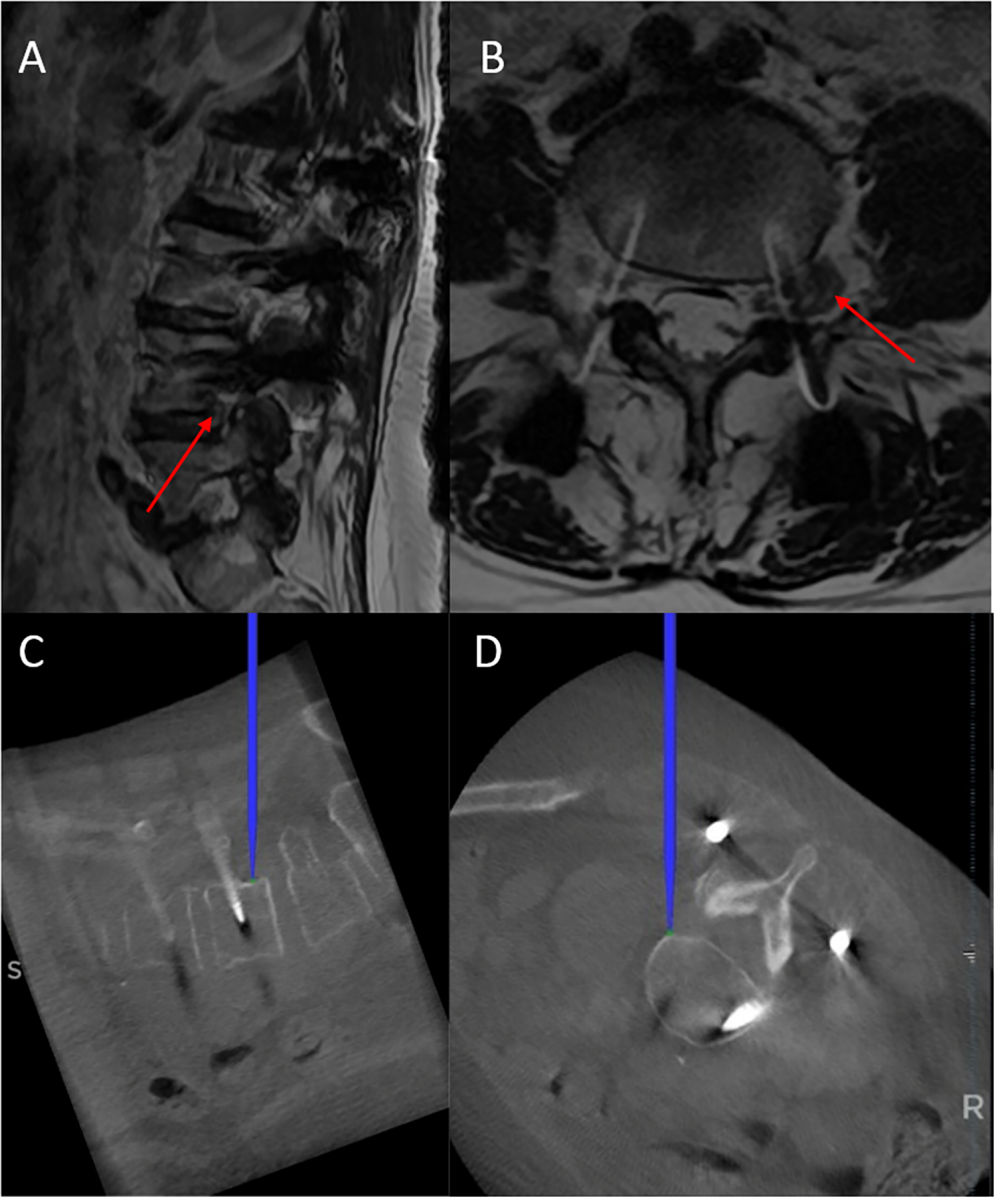
Upper: pre-operative T2-WI **(A)** sagittal and **(B)** axial MRI. Disc herniation at left L4–L5 foraminal-extraforaminal localization just beneath pedicle screw was evident (red arrow). Lower: **(C)** sagittal and **(D)** axial intraoperative CT-navigation showing the tip of the navigation probe positioned at the level of extruded fragment, allowing its rapid identification and removal.

A 59-years-old female patient presented at our outpatient clinic with a recent history of left sciatic pain, unresponsive to FANS and corticosteroid therapy. Nine years before she had undergone a decompression and pedicle-screws stabilization surgery at levels L2–L4. Lumbar spine MRI showed a foraminal–extra foraminal disc herniation at the adjacent level L4–L5 on the left (Figures 4A,B). At neurologic examination, she presented tactile hypoestesia with L4 distribution (8/10) and a positive left straight leg sign. In consideration of the extension of the disc herniation at extraforaminal area and the previous surgery a Wiltse approach was chosen, and we decided to use O-arm CT guided neuronavigation to perform it. After intraoperative CT acquisition the left L4–L5 foramen was easily addressed with the aid of neuronavigation (Figures 4C,D), the herniated fragment removed and the L4 left nerve completely decompressed. The operative time was 75 min and blood loss 80 ml, respectively. The post-operative course was uneventful with a complete recovery from the pain.

## Discussion

The Wiltse approach is a conventional anatomical approach to the far lateral spine compartment carried out through a dissection of medial multifidus and lateral longissimus muscles (4). It can be useful to treat different conditions such as intra-extraforaminal disc herniations, spinal stenosis, spondylolisthesis and spinal tumors. As previously reported, its main advantages include low intraoperative bleeding, shorter hospital length of stay, and a low infection rate compared with a standard midline approach (5). A previous study on posterior lumbar interbody fusion with pedicle screws and interbody cages at the L4–L5 level for lumbar degenerative spondylolisthesis comparing the midline approach or miniopen technique (bilateral Wiltse approach) evidenced no difference between these two approaches in term of clinical outcome but a reduced multifidus atrophy with the Wiltse approach (6). Furthermore, no differences in terms of complications, namely dural tears, hardware malposition, nerve root injury, or inadequate decompression, between these two approaches were reported when performed by experienced spine surgeons (5–23 years of experience) (5). On the other hand, the Wiltse approach may be limited by the need for familiarity with the anatomy (2). Further, the potential poor visibility in the Wiltse approach can, at least in theory, determine the risk of nerve root injury and other complications and make difficult the disk enucleation, especially when performed by young neurosurgeons (2). It has recently underlined that the Wiltse approach can be considered as a minimally invasive approach (1) and that a significant learning curve exists when approaching a minimally invasive spine technique for decompression or fusion exposing patients to potential complications and/or unsatisfactory results (7). Thus, the introduction of neuronavigation in this clinical scenario may provide valuable assistance to surgeons who are performing unfamiliar surgery or when a conventional procedure is complicated by anatomical alterations due to previous surgery and/or anatomic variations. Moreover it has been previously reported its utility in the fusion surgery (8, 9). This technical note provides a focus on how intraoperative CT-neuronavigation can help neurosurgeon in Wiltse approach. We used this approach to treat a dumbbell lumbar tumor and an intra-extraforaminal lumbar disc herniation adjacent to previous pedicle-screw stabilization. The final aim of using intraoperative CT-neuronavigation is to tailor the surgical approach in order to localize precisely the pathology, finally reducing soft-tissue trauma, complications and operative time. As for any procedure that involves CT-neuronavigation, the placement of the patient anchored reference is the first source of error. It must be placed on the closest spinous process available with respect to the surgical level to increase navigation accuracy. Care must be taken to securely verify its tightness on the spinous process bone, as any toggling may significantly affect accuracy. Before starting the procedure, the accuracy of navigation should be verified: fixed bone surfaces are ideal, thus the tip of the spinous process where the reference is anchored may serve for this purpose (10). Skin incision can then be planned. The employment of CT-neuronavigation allowed us to tailor the extension and position of skin incision as well as the extension of muscles dissection and to perfectly center the approach over the intervertebral foramen of interest (11). In both cases, we were able to precisely localize the lesions. In the case of schwannoma we were able to demarcate the limits of tumor, particularly the lateral edge, avoiding unnecessary traction of lumbar plexus and iliopsoas muscle (see Figure 1). Particularly in the case of intra-extraforaminal disc herniation, the planning of the right trajectory was greatly facilitated by CT-neuronavigation, since the tip of the navigation probe was perfectly centered over the extruded fragment which migrated cranially along the posterior border of the L4 vertebral body being placed caudally to the ipsilateral pedicle screw and ventral to the rod (see Figure 4).

The main limitation of this paper is that it is based on two successful cases and general conclusions on the effective advantage of application of CT-neuronavigation to Wiltse approach cannot be taken. Further, we were not able to calculate the radiation exposure in these two cases.

In conclusion, this technical note explores the application of intraoperative CT-neuronavigation to the Wiltse approach. We performed this technique in two cases, an intra-extraforaminal lumbar schwannoma and an extraforaminal disc herniation in a level adjacent to a previous pedicle screw stabilization. We were able to easily identify and remove both the lesions minimizing the surgical approach with no complication and optimal clinical outcome. Further studies are needed to address the effective utility and advantages of application of intraoperative CT-neuronavigation to Wiltse approach.

## Data Availability

The original contributions presented in the study are included in the article/Supplementary Material, further inquiries can be directed to the corresponding author.
